# Workplace Incivility and Nurses' Health: The Mediating Role of Positive and Negative Affect

**DOI:** 10.1002/nur.70063

**Published:** 2026-02-26

**Authors:** Ju Yeon Lee, Sujin Nam, Heejin Lim, Chang Gi Park, Seung Eun Lee

**Affiliations:** ^1^ Department of Psychology Yonsei University Seoul South Korea; ^2^ College of Nursing Yonsei University Seoul South Korea; ^3^ College of Nursing University of Illinois Chicago Chicago Illinois USA; ^4^ Mo‐Im KIM Nursing Research Institute, College of Nursing Yonsei University Seoul South Korea

**Keywords:** affect, incivility, mediation analysis, nurses, self‐rated health

## Abstract

Nurses are particularly vulnerable to workplace incivility, a form of deviant behavior that can evolve into severe workplace mistreatment. Although the psychological and organizational consequences of incivility have been documented, its impact on health, particularly through emotional pathways, remains unclear. Therefore, this study investigated the association between workplace incivility and nurses' self‐rated health, focusing on the mediating roles of positive and negative affect—broad emotional states reflecting energy and engagement, and distress and unpleasant emotions, respectively. This descriptive correlational study used survey data from 301 nurses in South Korea. Generalized structural equation modeling was employed to test the hypothesized relationships between workplace incivility, affective responses, and health. Workplace incivility was significantly associated with reduced positive affect (*b* = −0.41, *p* < 0.001) and increased negative affect (*b* = 2.78, *p* < 0.001). While positive affect was positively related to self‐rated health (*b* = 1.58, *p* < 0.001), negative affect was negatively associated (*b* = −0.50, *p* = 0.017). Both affective responses significantly mediated the relationship between workplace incivility and health (indirect effects: −0.65, *p* < 0.001; −1.38, *p* = 0.022, respectively). Even subtle forms of incivility can adversely relate to nurses' self‐rated health through emotional states, such as reduced positive affect and increased negative affect. Fostering a respectful workplace and promoting emotional well‐being may support nurses' health. These findings extend affective events theory to health outcomes, an area rarely explored in nursing research. No patients or the general public were involved.

## 1 Introduction

Workplace incivility is an increasingly recognized problem in the healthcare industry (Freedman et al. 2024), particularly in nursing, where high‐stress environments and complex interpersonal dynamics often lead to interpersonal tension and conflict (Jackson et al. 2024). Incivility refers to relatively mild forms of disrespectful or inappropriate behavior that may not clearly aim to harm but still breach expected standards of mutual respect among employees (Andersson and Pearson 1999). Although such behaviors may seem insignificant in isolation, they can cumulatively damage the work environment and employee well‐being. It is often subtle, covert, and difficult to detect (Schilpzand et al. 2016). In nursing contexts, it may take the form of gossip, social exclusion, and undermining behaviors (Alsadaan et al. 2024).

Despite its subtlety, workplace incivility is alarmingly prevalent. A recent meta‐analysis showed that nearly a quarter of nurses have personally experienced incivility, and over a third have witnessed it (Freedman et al. 2024). Though there are distinct forms of workplace aggression, including verbal or physical violence (National Institute for Occupational Safety and Health 2022), incivility is considered a continuum of workplace mistreatment (Bambi et al. 2018). If left unaddressed, it can escalate into more severe forms of aggression (National Institute for Occupational Safety and Health 2022), contribute to prolonged workplace mistreatment (Holm et al. 2022), and reinforce a negative organizational climate (Zahid and Nauman 2024).

Although workplace incivility has been linked to a variety of negative outcomes for nurses (Bambi et al. 2018), most existing research has focused on psychological or occupational impacts. A scoping review highlighted a lack of attention to health outcomes in healthcare settings (Martin and Zadinsky 2022). The few studies that have examined this connection focus primarily on nursing faculty members (Sherrod and Lewallen 2021), new graduate nurses (Read and Laschinger 2013), or nonhealthcare workers (Hasson and Villaume 2024), leaving clinical nurses underrepresented. This gap is particularly concerning given the well‐established relationship between workplace violence and adverse health outcomes among nurses (Magnavita et al. 2022; Pérez‐Fuentes et al. 2020; Zhang et al. 2025). As a more subtle but prevalent form of mistreatment, incivility may similarly undermine nurses' health. It is therefore essential to investigate whether incivility is associated with nurses' health and identify its mechanisms.

Although existing research has linked workplace violence to nurses' health outcomes (Feruglio et al. 2025), it has largely focused on direct associations, often overlooking the psychological processes that may underlie these effects. Affective events theory (AET; Weiss and Cropanzano 1996) provides a useful framework for addressing this gap: workplace events influence individual outcomes primarily through emotional responses they elicit rather than through direct pathways. In the context of incivility, such responses may include decreases in positive affect, such as enthusiasm (Hasson and Villaume 2024), and increases in negative affect, such as anger (Anjali and Nemat Sheereen 2024; Tremmel and Sonnentag 2018), or both (Giumetti et al. 2013). These affective states, in turn, offer a plausible mechanism linking workplace incivility to health‐related outcomes, particularly to how individuals perceive and evaluate their health.

There is convergent evidence from multiple studies indicating that affect precedes and shapes health outcomes. For instance, longitudinal analyses show that positive affect is associated with later reductions in inflammatory markers, independent of mental health status (e.g., Fancourt and Steptoe 2020). Meta‐analytic evidence also indicates that positive affect is linked to a reduced risk of subsequent mortality (Chida and Steptoe 2008). To address potential reverse causality, experimental evidence demonstrates that negative affect predicts subsequent cortisol levels, while the reverse pathway (i.e., cortisol predicting negative affect) is non‐significant (Adam et al. 2006). Notably, comprehensive reviews reveal that both positive and negative affect are linked to self‐rated health and broader health outcomes, including longevity (Diener and Chan 2011) and cardiovascular health (Boehm and Kubzansky 2012). Additionally, emotional styles have been associated with subjective symptom reporting independent of objective infection status (Cohen et al. 2003). Based on this evidence, it is reasonable to hypothesize that both positive and negative affect relate to individuals' perceptions and evaluations of their health.

Positive and negative affect play distinct roles in shaping individuals' evaluations of their health. Positive affect is associated with better self‐rated health, partly through its role in buffering stress and fostering psychological resilience (Fredrickson 2001). Based on the broaden‐and‐build theory (Fredrickson 1998, 2001), such affective states promote resource accumulation and adaptive coping. On the other hand, negative affect has been linked to physiological dysregulation (Graham‐Engeland et al. 2018; Pauly et al. 2021), which may lead to greater somatic complaints (Watson and Pennebaker 1989) and more negative health appraisals (Finch et al. 2012). These findings support the notion that affective states may serve as key psychological pathways connecting workplace incivility to perceived health.

Conservation of resources (COR) theory (Hobfoll 1989, 2001) further explains the emotional toll of incivility. This theory posits that stress and psychological distress emerge when individuals lose or fear losing valuable resources—such as emotional energy, cognitive capacity, or time—and when coping itself consumes those same resources (Hobfoll and London 1986). Although often considered less severe than overt aggression, incivility's chronic and persistent nature allows it to function as a daily micro‐stressor (Cortina et al. 2022). Over time, the accumulation of stressors can deplete internal resources, leading to shifts in emotional states (Zhou et al. 2015). Empirical evidence supports this mechanism, showing that persistent incivility reduces positive emotions (Hasson and Villaume 2024) and frequent exposure increases negative emotions (Sakurai and Jex 2012).

To fully capture the emotional mechanisms underlying the relationship between workplace incivility and health, it is important to model positive and negative affect as distinct yet co‐occurring constructs. Rather than existing on the opposite ends of a single continuum, these affective states function as independent dimensions (Zautra et al. 1997). The lack of positive affect does not imply the existence of negative affect, nor does low negative affect indicate high positive affect (Diener and Emmons 1984). A single adverse event can simultaneously reduce positive affect and increase negative affect (Cacioppo and Berntson 1994), such as workplace incivility, making it essential to examine both concurrently. However, many prior studies have modeled them in isolation, limiting insights into their differential and combined effects (Finch et al. 2012). Thus, the present study addresses this limitation by modeling both positive and negative affect as concurrent mediators.

Drawing on the AET and COR theory, this study conceptualizes positive and negative affect as distinct but concurrent mediators linking workplace incivility to self‐rated health among nurses. We proposed the following hypotheses:

Hypothesis 1 Workplace incivility is negatively associated with positive affect and positively associated with negative affect.

Hypothesis 2 Positive affect is positively associated with self‐rated health, whereas negative affect is negatively associated with self‐rated health.

Hypothesis 3 Both positive and negative affect mediate the relationship between workplace incivility and self‐rated health.

## 2 Methods

### 2.1 Design and Setting

This descriptive correlational study used data from the first wave of a larger longitudinal survey of registered nurses in South Korea. Participants were recruited through a widely used online community for Korean nurses and completed online survey (Qualtrics XM, Provo, UT, USA) between December 2024 and February 2025. Inclusion criteria were as follows: (1) being a registered nurse currently employed in a hospital unit providing direct nursing care to patients and (2) having at least 6 months of clinical experience at their current hospital, as this is the minimum duration required to gauge workplace incivility (Carmona‐Cobo and Lopez‐Zafra 2022; Kim and Lee 2024). Eligibility was screened at survey start, and those not indicating a direct nursing‐care registered nurse role could not proceed. The participants were encouraged to share the survey link with other potentially eligible colleagues. Due to the open‐link and online recruitment method, the exact response rate could not be calculated. Of 396 accesses, 95 cases were excluded for providing only minimal demographics (no exposure/mediator/outcome data) or for careless responding (e.g., straight‐lining), yielding an analytic sample of 301 with complete data. This final sample size exceeded the generally recommended minimum of 200 for structural equation modeling (Harrington 2009) and was therefore considered sufficient for the planned analyses.

### 2.2 Measure

#### 2.2.1 Dependent Variable

Self‐rated health was assessed with a single general health item from the Short‐Form Health Survey (SF‐12; Ware et al. 1996): “In general, would you say your health is?” This item is widely used in health and well‐being research (e.g., National Institute for Occupational Safety and Health 2021). Participants rated their general health on a 5‐point scale ranging from 1 (*poor*) to 5 (*excellent*), with higher scores indicating better perceived health. This single‐item measure is a widely accepted and reliable measurement across various populations (Molina et al. 2019).

#### 2.2.2 Independent Variable

Workplace incivility was measured using seven items from the Workplace Incivility Scale, which has shown good psychometric properties (Cortina et al. 2001) and has been used for the Korean nurse population (Lee et al. 2024). An example item is “Have you been in a situation where any of your superiors or coworkers made demeaning or derogatory remarks about you?” Participants rated the frequency of incivility experiences on a 5‐point scale ranging from 1 (*never*) to 5 (*most of the time*), with higher scores indicating more frequent incivility. In the present study, Cronbach's *α* coefficient was 0.91.

#### 2.2.3 Mediators

Positive and negative affect were assessed using the Positive and Negative Affect Schedule (Watson et al. 1988), which has been translated and validated for the Korean population (Park and Lee 2016). Each subscale consists of 10 adjectives describing emotional states. Participants rated how much they experienced each emotion on a 5‐point scale ranging from 1 (*not at all*) to 5 (*extremely*). Example adjectives include “interested” (positive affect) and “irritable” (negative affect). Higher scores indicate a greater tendency to experience the respective affective states. In the present study, Cronbach's *α* was 0.93 for both subscales.

### 2.3 Statistical Analysis

Descriptive statistics were computed to summarize participant characteristics and key study variables. Bivariate relationships were examined using both Pearson's and Spearman's correlation coefficients. Prior to the main analyses, we conducted confirmatory factor analysis (CFA) in R (version 4.4.1) with the weighted least squares means and variance adjusted (WLSMV) estimator to evaluate the construct validity and factor structure of the workplace incivility and affect scales. The CFA was performed solely to assess measurement quality. For the main analyses, composite scores were calculated by averaging the items for workplace incivility, positive affect, and negative affect. These composite scores were treated as observed variables in the structural models rather than modeling them as latent variables, so the structural component of the analysis is a path analysis with observed composite variables. Because the dependent variable, self‐rated health, was measured on an ordinal scale, we estimated this path model using generalized structural equation modeling (GSEM) with a logit link function (StataCorp 2025). Age, unit tenure (Pace et al. 2023), and hospital type (Haro‐Ramos et al. 2024) were included as control variables based on previous research indicating their association with nurses' health ratings. To examine the indirect effects of workplace incivility on self‐rated health, a mediation analysis was conducted using 5000 bootstrap replications to obtain robust standard errors and confidence intervals (CI). Except for CFA, all analyses were conducted using Stata 19.5, with significance set at *p* < 0.05.

### 2.4 Ethical Consideration

This study involving human participants received ethical approval from the Institutional Review Board of the Yonsei University Health System (#4‐2025‐0417). This research procedures complied with the ethical principles established by the Declaration of Helsinki. All participants provided informed consent prior to participation. Participation was voluntary and anonymous, and the obtained data were confidential. The participants were informed of their right to withdraw from the study at any time without a penalty.

## 3 Results

### 3.1 Sample Characteristics and Bivariate Correlations

As shown in Table 1, most participants were women (96.0%), with a mean age of 30.74 years (SD = 4.67). On average, participants had 5.75 years (SD = 4.40) of total nursing experience, 4.94 years (SD = 4.03) of tenure at their current hospital, and 3.35 years (SD = 2.21) of tenure in their current unit. Most participants were full‐time employees working in either public (25.2%) or private (74.8%) hospitals.

**Table 1:** Participants' demographic and occupational characteristics (*N* = 301).

Variable	Category	*n*	%	Mean	SD
Age (years)				30.74	4.67
Sex	Female	289	96.0		
	Male	12	4.0		
Clinical experience (years)				5.75	4.40
Tenure at current hospital (years)				4.94	4.03
Tenure at current unit (years)				3.35	2.21
Current working unit	General medicine	256	85.0		
	Specialty unit	29	9.6		
	Other	16	5.4		
Hospital type	Private	225	74.8		
	Public	76	25.2		

Abbreviation: SD, standard deviation.

As shown in Table 2, workplace incivility negatively correlated with positive affect (Pearson's *r* = −0.19, *p* < 0.001) and positively correlated with negative affect (Pearson's *r* = 0.78, *p* < 0.001). According to Rönkkö and Cho (2022), correlations below 0.80 generally indicate no concerns regarding discriminant validity. Thus, although the correlation between workplace incivility and negative affect is relatively strong, it remains within the acceptable range. Positive affect positively correlated with self‐rated health (Spearman's *r* = 0.66, *p* < 0.001), and negative affect negatively correlated with self‐rated health (Spearman's *r* = −0.41, *p* < 0.001).

**Table 2:** Mean, standard deviation, and correlations among the study variables (*N* = 301).

Variable	1	2	3	4
1. Workplace incivility	—			
2. Positive affect	−0.19*** ^,^ a	—		
3. Negative affect	0.78*** ^,^ a	−0.34*** ^,^ a	—	
4. Self‐rated health	−0.26*** ^,^ b	0.66*** ^,^ b	−0.41*** ^,^ b	—
Mean	1.83	3.45	2.09	—
Standard deviation	0.84	0.97	0.92	—

^a^ Pearson's correlation coefficient.

^b^ Spearman's rho correlation coefficient.

*** *p *< 0.001.

### 3.2 Path Analysis

The CFA indicated good fit to the data—*χ* ^2^(345) = 423.063, comparative fit index (CFI) = 0.993, Tucker–Lewis index (TLI) = 0.992, root mean square error of approximation (RMSEA) = 0.027, standardized root mean squared residual (SRMR) = 0.067—with all factor loadings significant (Hair et al. 2019). These findings support the construct validity of the workplace incivility and affect scales and justify the use of composite scores in the subsequent GSEM‐based path analyses. Our path analysis using observed composite scores demonstrated that workplace incivility was significantly associated with positive and negative affect, and both affect dimensions were significantly associated with self‐rated health (Figure 1). Specifically, higher workplace incivility scores were linked to reduced positive affect (*b* = −0.41, *p* < 0.001; odds ratio [OR] = 0.66, 95% CI: [0.55, 0.82]) and increased negative affect (*b* = 2.78, *p* < 0.001; OR = 16.05, 95% CI: [11.69, 26.99]). The affect variables, in turn, significantly predicted self‐rated health. Specifically, positive affect was positively associated with higher self‐rated health (*b* = 1.58, *p* < 0.001; OR = 4.86, 95% CI: [3.87, 5.94]), while negative affect was negatively associated with lower self‐rated health (*b* = −0.50, *p* = 0.017, OR = 0.61, 95% CI: [0.45, 0.90]).

**Figure 1:**
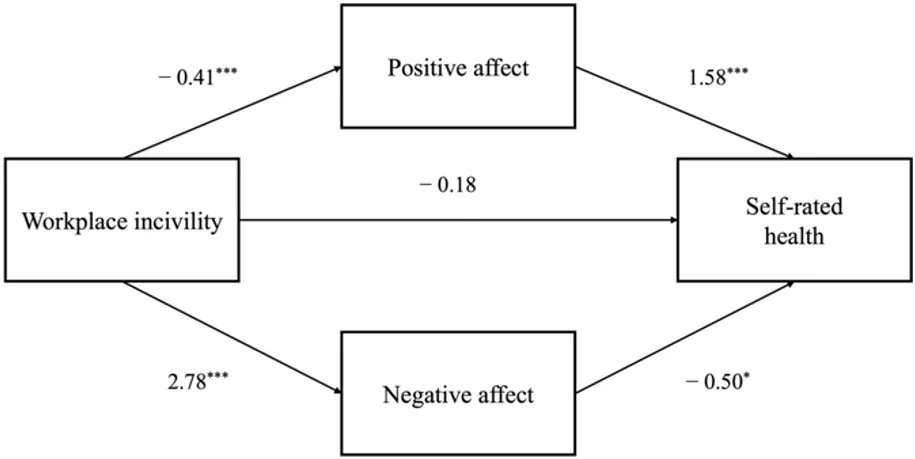
Path diagram of the mediation model. Generalized structural equation modeling (GSEM) path model with observed composite variables (rectangles) linking workplace incivility to self‐rated health via positive and negative affect. Self‐rated health was modeled as an ordinal outcome with a logit link. Values are unstandardized coefficients; paths into self‐rated health are log‐odds. Models adjust for age, unit tenure, and hospital type (paths omitted for clarity). ***p < 0.001, *p < 0.05.

To examine the mediating roles of positive and negative affect, a bootstrapped mediation analysis with 5000 replications was conducted to estimate the indirect effects and obtain bias‐corrected 95% CIs. As shown in Table 3, the total effect of workplace incivility on self‐rated health was significant (total effect = −2.20, SE = 0.48, *p* < 0.001, OR = 0.11, 95% CI: [0.04, 0.28]). Conversely, the direct effect of workplace incivility on self‐rated health was not statistically significant (direct effect = −0.18, SE = 0.25, *p *= 0.477; OR = 0.84, 95% CI: [0.51, 1.24]). Positive affect significantly mediated the relationship between workplace incivility and self‐rated health, with an indirect effect of −0.65 on a logit scale (SE = 0.18, *p* < 0.001, OR = 0.52, 95% CI: [0.38, 0.73]). This finding suggests that workplace incivility may be associated with poorer self‐rated health through its relationship with positive affect. Likewise, negative affect significantly mediated the relationship between workplace incivility and self‐rated health (indirect effect = −1.38, SE = 0.60, *p* = 0.022, OR = 0.25, 95% CI: [0.09, 0.77]), suggesting that increased negative affect may be linked to the association between workplace incivility and poorer self‐rated health. The total effect of workplace incivility on self‐rated health was transmitted primarily through indirect pathways via positive and negative affect, as the direct effect was not statistically significant. These results suggest that affective states may play an important mediating role in this association.

**Table 3:** Total, direct, and indirect effects in the hypothesized path model (*N* = 301).

Path	*b*	SE	*p*	OR	95% CI	
					Lower	Upper
Total effect						
Workplace incivility → self‐rated health	−2.20	0.48	< 0.001	0.11	0.04	0.28
Direct effect						
Workplace incivility → self‐rated health	−0.18	0.25	0.477	0.84	0.51	1.24
Indirect effect						
Workplace incivility → positive affect → self‐rated health	−0.65	0.18	< 0.001	0.52	0.38	0.73
Workplace incivility → negative affect → self‐rated health	−1.38	0.60	0.022	0.25	0.09	0.77

Abbreviations: b, unstandardized coefficient; CI, bias‐corrected confidence interval; OR, odds ratio; SE, standard error.

## 4 Discussion

This study examined whether positive and negative affect mediate the relationship between workplace incivility and self‐rated health among nurses, drawing on AET and COR theory. The findings revealed that affective responses played a mediating role in this relationship. Specifically, the results suggest that emotional mechanisms are associated with how workplace incivility relates to perceived health in a clinical nursing context.

As expected, workplace incivility was associated with lower positive affect and higher negative affect, supporting Hypothesis 1. These findings align with prior research in nonhealthcare settings showing that exposure to incivility is linked to reduced positive affect (Hasson and Villaume 2024), increased negative affect (Anjali and Nemat Sheereen 2024; Tremmel and Sonnentag 2018), or both (Giumetti et al. 2013). By replicating these associations, our study aligns with prior findings and extends them to the nursing context. Furthermore, these results reinforce the fundamental proposition of AET that discrete workplace events can trigger emotional responses (Weiss and Cropanzano 1996). Additionally, consistent with COR theory (Hobfoll 1989, 2001), workplace incivility appears to threaten individuals' emotional resources, contributing to psychological strain (Parray et al. 2023). The observed reductions in positive affect and increases in negative affect may reflect the emotional toll of ongoing resource loss. These findings are consistent with a theory‐driven account in which incivility is associated with nurses' emotional responses.

In support of Hypothesis 2, positive affect was positively associated with self‐rated health, whereas negative affect was linked to poorer health appraisals. These findings align with the broaden‐and‐build theory (Fredrickson 1998, 2001), which suggests that positive affect broadens cognitive and emotional resources and fosters more favorable health perceptions. Beyond these theoretical accounts, our findings echo evidence that affective states precede subsequent health outcomes in longitudinal, meta‐analytic, and experimental work (Adam et al. 2006; Chida and Steptoe 2008; Fancourt and Steptoe 2020). Specifically, our findings are consistent with previous research showing that positive affect is associated with more positive health appraisals (Nath and Pradhan 2012), whereas negative affect contributes to poorer health perceptions (O'Leary et al. 2021), possibly due to its physiological burden (DeSteno et al. 2013). Examining these associations in a sample of nurses—who frequently face emotional and physical demands—adds occupational relevance to previous findings. Taken together, the results align with broader evidence that affective states are associated with health perceptions and provide nursing‐specific evidence consistent with an affective pathway.

Hypothesis 3 was also supported: both positive and negative affect showed significant indirect associations in the relationship between workplace incivility and self‐rated health among nurses. Building on prior research linking workplace violence and adverse health outcomes (Magnavita et al. 2022; Pérez‐Fuentes et al. 2020; Zhang et al. 2025), our findings demonstrated that even lower‐intensity mistreatment, such as incivility, is negatively associated with perceived health via emotional mechanisms. Notably, the direct association between workplace incivility and self‐rated health was not statistically significant, whereas both affective pathways showed significant indirect effects. This pattern suggests that positive and negative affect may play a central role in linking workplace incivility to health perceptions, although these findings should not be interpreted as evidence of complete mediation. Rather, they highlight affective processes as important explanatory mechanisms in this association. This finding is consistent with previous research on workplace bullying, another form of workplace violence (NIOSH 2022), where negative emotions (Ciby and Raya 2018) and emotional exhaustion (Liang 2021) were identified as partial mediators of health outcomes. It provides robust support for AET, affirming that emotional reactions are a key pathway through which workplace mistreatment is related to attitudinal and behavioral outcomes in nursing (Christensen et al. 2023). By demonstrating a similar mechanism in the context of incivility, our results align with AET and extend its application to self‐rated health in nursing.

A notable contribution of this study lies in its simultaneous examination of both positive and negative affect within a single model, an approach that remains relatively uncommon in the existing literature. This methodological decision was grounded in evidence indicating that positive and negative affect are not diametrically opposed but operate as independent dimensions (Zautra et al. 1997). By including both in a single model, we identified their unique and concurrent mediating roles. The fact that both remained significant mediators when tested together supports the idea that these emotional states are independently activated and show distinct associations (Eryilmaz et al. 2023). This is especially important given that many prior studies examined these dimensions in isolation, potentially obscuring their differentiated contributions (Finch et al. 2012). Our study provides a more nuanced understanding of the emotional mechanisms linking workplace incivility to self‐rated health.

Our findings suggest important practical implications for healthcare organizations aiming to improve nurses' well‐being. Although often subtle, workplace incivility is associated with poorer self‐rated health through emotional states. Educational initiatives should raise awareness of the cumulative effects of incivility, not only on psychological outcomes but also on health, and offer practical strategies for managing such behavior. For instance, cognitive rehearsal training has been shown to help nurses respond effectively to uncivil encounters (Givler and Varagona 2024). Nurse managers also play a crucial role in fostering positive affect by promoting supportive team practices and peer‐based support, which have been linked to greater resilience (Connors et al. 2024). Given the link between incivility and increased negative affect, early recognition and assessment of emotional distress are essential. A recent meta‐analysis (Ong et al. 2024) highlights the effectiveness of workplace interventions, such as mindfulness training, in reducing stress, anxiety, and depression among healthcare workers. Moreover, institutional policies should explicitly address workplace incivility by establishing clear behavioral expectations, enabling anonymous reporting, and enforcing accountability. Research suggests that such approaches can be effective when they comprehensively tackle both individual and systemic contributors to incivility (Maben et al. 2023). Collectively, these strategies may help reduce the emotional burden of incivility, improve nurses' health outcomes, and promote a more sustainable and supportive work environment.

### 4.1 Limitations

This study has some limitations that should be considered. First, its cross‐sectional design precludes establishing temporal precedence and leaves open the possibility of reverse causation. Thus, our findings should be interpreted as theory‐consistent associations rather than causal effects. Future work using multi‐wave longitudinal designs is recommended. Second, the use of a single‐item measure may not fully capture the multidimensional nature of an individual's health status, although it is a globally recognized and considered a reliable indicator in health and well‐being research (Molina et al. 2019). Third, several potentially relevant variables were not included in the analysis. Factors such as job stress (Dua 1994), social support (Hale et al. 2005; Scholz et al. 2012), and emotional intelligence (Kafetsios and Zampetakis 2008; Martins et al. 2010) may influence both affective responses and self‐evaluation of health. Their omission may limit the completeness of the proposed model and should be addressed in future research. In addition, we conducted a path analysis using observed composite variables, which does not explicitly model measurement error and may potentially attenuate the estimated path coefficients. Finally, because the participants were recruited from a single online nursing community, the findings may not be generalizable to the broader nursing population in South Korea. Future studies should investigate whether similar patterns exist across different clinical settings, professional roles, and cultural contexts.

## 5 Conclusions

This study investigated the relationship between workplace incivility and self‐rated health among nurses, focusing on the mediating roles of both positive and negative affect. Our findings demonstrate that even low‐intensity mistreatment in the workplace can have significant health implications through one's emotional state. These results underscore the importance of organizational strategies that address emotional responses and promote respectful workplace environments. This study provides valuable insights into the emotional pathways through which incivility may be related to health. Future research should investigate whether these patterns hold across different occupational groups and cultural contexts, thereby improving the applicability of the results to broader populations.

## Author Contributions

**Ju Yeon Lee:** conceptualization, writing – original draft, writing – review and editing. **Sujin Nam:** conceptualization, formal analysis, writing – original draft, writing ‐ review and editing. **Heejin Lim:** conceptualization, investigation, data curation, writing – original draft, writing – review and editing, project administration. **Chang Gi Park:** conceptualization, formal analysis, writing – review and editing. **Seung Eun Lee:** conceptualization, methodology, formal analysis, investigation, writing – original draft, writing – review and editing, supervision, funding acquisition.

## Conflicts of Interest

The authors declare no conflicts of interest.

## Data Availability

The data that support the findings of this study are available on request from the corresponding author. The data are not publicly available due to privacy or ethical restrictions.
